# Live *Tenebrio molitor* larvae as a dietary supplement for post-weaning piglets: effects on performance and health under diets with different crude protein levels

**DOI:** 10.3389/fvets.2026.1797385

**Published:** 2026-03-18

**Authors:** Luciana Rossi, Sara Frazzini, Matilda Rachele Dametti, Camilla Dudiez, Sara Barbieri, Roberto Pilu, Elena Cassani, Vincenzo Chiofalo, Matteo Dell’Anno

**Affiliations:** 1 Department of Veterinary Medicine and Animal Sciences (DIVAS), University of Milan, Milan, Italy; 2 Department of Agricultural and Environmental Sciences – Production, Landscape, Agroenergy (DiSAA), University of Milan, Milan, Italy; 3 Department of Veterinary Sciences, University of Messina, Messina, Italy

**Keywords:** alternative protein sources, crude protein reduction, environmental enrichment, live mealworm larvae, post weaning diarrhoea, sustainable nutrition, weaning pigs

## Abstract

**Introduction:**

Weaning represents one of the most critical phases in pig production, as it is often characterised by a transient period of fasting that can impair gut integrity and ultimately reduce growth performance while increasing susceptibility to disease. At the same time, increasing pressure to reduce dietary crude protein and reliance on conventional protein sources has highlighted the need for feeding strategies that support the sustainability of feed formulations and animal health.

**Aim:**

This study evaluated the effects of daily supplementation with live *Tenebrio molitor* larvae on performance, feed intake, health status, digestibility and metabolic markers in post-weaning piglets fed diets containing moderate (15%) or standard (17%) crude protein levels.

**Methods:**

A total of 48 piglets were allocated to four treatments: control diets without larvae (CTRL15 and CTRL17) and corresponding diets supplemented daily with 50 g/pig/day of live mealworm larvae (INS15 and INS17). Growth performance, feed intake, feed efficiency, time required to consume the larvae, health indicators, apparent total tract digestibility, serum biochemical parameters, antioxidant capacity and mineral profile were assessed over a 42-day trial.

**Results and discussion:**

Larvae supplementation significantly improved early feed efficiency and growth performance (*p* < 0.05), particularly during the first weeks post-weaning. Piglets in INS17 achieved higher final body weight compared with CTRL groups (*p* < 0.05), while INS15 piglets reached performance comparable to CTRL17, despite the lower dietary protein level. Live larvae were rapidly and consistently consumed from the moment of supplementation, with consumption time decreasing below 2 min during days 30–42 of the trial, whereas soybean meal supplementation elicited lower feeding interest in piglets with longer consumption time. Larvae supplementation was associated with an improved health status, including a lower incidence of diarrhoea, reduced occurrence of respiratory disorders and higher vitality scores compared with control groups (*p* < 0.05) throughout the trial. No differences were observed in nutrient digestibility, serum metabolic profile and antioxidant status at day 42.

**Conclusion:**

In conclusion, live *T. molitor* larvae represent a promising functional feeding strategy to stimulate early feed intake during weaning and enhance piglet resilience, supporting growth performance and health while allowing dietary protein levels to be moderately reduced without negative effects.

## 1 Introduction

The immediate post-weaning period represents a critical phase for piglets, characterized by a marked reduction in feed intake, reduced digestive efficiency due to gastrointestinal immaturity, and increased susceptibility to multifactorial gastrointestinal disorders (1).

During this period, piglets are exposed to concurrent nutritional, environmental, and social stressors associated with weaning, and the abrupt transition from highly digestible sow’s milk to solid feed may induce transient anorexia (2–4). These stress-related changes can reduce nutrient availability to the intestinal mucosa, promoting structural and functional alterations such as villous atrophy, shifts in the intestinal microbiota, and metabolic disturbances such as impaired glycaemic regulation (5, 6). Early feed intake has therefore been identified as a key factor of post-weaning resilience, with animals identified as “early eaters” typically exhibiting improved growth performance during the critical days following weaning (7).

This aspect is particularly relevant in the broader context of antimicrobial resistance, which represents a major global threat to both animal and public health. The promotion of antimicrobial stewardship in livestock calls for strategies aimed at preventing disease and reducing the reliance on antimicrobial treatments (8). In this framework, promoting gut stability and limiting post-weaning disorders through nutritional approaches can contribute to lowering the need for antimicrobial interventions, in line with One Health principles (9). For these reasons, dietary management during the post-weaning period plays a crucial role in maintaining gut health, as gastrointestinal disorders remain among the leading causes of antimicrobial use in pig production (10). In this context, diet palatability is of primary importance in limiting the extent and duration of post-weaning anorexia, thereby promoting an early recovery of feed intake and supporting the supply of essential nutrients to the gastrointestinal tract (2).

Beyond feed acceptance, the digestibility and biological quality of dietary protein are critical for ensuring efficient nutrient utilization, supporting growth performance and health status, and limiting nitrogen excretion (11, 12).

In parallel, the livestock sector is facing increasing pressure to adopt more sustainable feeding strategies, decrease reliance on imported protein sources (13–15). The strong dependence of the European livestock sector on soybean meal, combined with fluctuations in its availability, cost, and environmental footprint, has intensified interest in alternative protein sources (16, 17). At the same time, there is a growing need to reduce reliance on traditional animal-derived proteins, such as fish meal, a commodity whose production is increasingly associated with significant environmental pressures (18, 19). These sustainability issues further highlight the importance of identifying sustainable nutrient inputs for pig nutrition.

Dietary crude protein (CP) level in post-weaning pig diets remains highly debated because it lies at the intersection of growth performance and gut health. Weaner diets are often formulated with relatively high CP to meet amino acid requirements under conditions of limited voluntary feed intake. However, weaning-associated constraints on protein digestion can increase the flow of undigested protein to the distal intestine, promoting proteolytic fermentation and the production of metabolites that negatively affect intestinal homeostasis (20). Accordingly, reduced-CP strategies have been proposed to limit fermentable nitrogen substrates and improve gut health, but their successful application depends on maintaining an adequate supply of digestible essential amino acids and overall protein quality to avoid impairments in growth performance (21).

Therefore, the identification of alternative protein sources capable of providing highly digestible, high biological value protein is essential to support optimized dietary CP levels while maintaining post-weaning performance.

In addition, the search for alternative protein sources is also driven by sustainability concerns, European Protein Gap, raw material price volatility, the reliance of many livestock production systems on imported plant-based proteins, and the need to diversify high-value feed ingredients to support sustainable livestock production (22–25).

In this global scenario, insects represent a promising option for animal nutrition due to their high biological value proteins with a favourable essential amino acid profile, their efficient bioconversion of organic substrates, their relatively low requirements for land and water compared with conventional protein crops, and the presence of antimicrobial peptides or other bioactive compounds that may confer additional functional properties (26, 27). Among insect species evaluated for animal feeding, the yellow mealworm (*Tenebrio molitor*) has received particular attention due to its nutritional composition, regulatory acceptance, and suitability for inclusion in pig diets (28, 29).

However, current research on *T. molitor* in pig nutrition has almost exclusively focused on processed forms such as dried larvae or insect meals, whereas information on the nutritional use of live larvae remains extremely limited. The provision of live larvae represents a distinct feeding strategy, characterized by differences in moisture content, physical form, and daily administration, which may influence nutrient intake, digestibility, and growth performance and therefore warrants specific investigation (30, 31). Studies in piglets have shown that live larvae can function as edible enrichment, stimulating exploratory and feeding behaviours that align with the pig’s natural motivation to root and manipulate substrates (32, 33). By providing a highly attractive, showing active movement feed item, live larvae may enhance feeding motivation, reduce stress-driven behaviours, and promote earlier and more consistent feed intake during the post-weaning period (32). These functional attributes are particularly relevant at a time when reduced intake can favour intestinal dysbiosis and growth impairment (2, 34).

Despite the mechanistic possibility and the growing interest in the use of insects as feed, scientific evidence regarding the use of live *T. molitor* larvae in weaned piglets remains very limited. Moreover, no studies have evaluated how the inclusion of live *T. molitor* larvae interacts with diets differing in crude protein content, an aspect of practical relevance for designing feeding strategies that support both intestinal health and sustainable protein management.

We hypothesised that the inclusion of live *T. molitor* larvae represents a biologically active dietary component capable of interacting with post-weaning physiological adaptation processes, and that such interaction may vary under diets differing in crude protein content. Accordingly, the aim of this study was to evaluate the effects of live *T. molitor* larvae supplementation on feed intake, growth performance, nutrient digestibility, blood metabolic profile, and health indicators in weaned piglets fed isoenergetic diets containing moderate (15%) or standard (17%) crude protein levels, using an isonitrogenous soybean meal supplementation as a control.

## 2 Materials and methods

### 2.1 *Tenebrio molitor* larvae rearing and preparation

*T. molitor* larvae were produced within a decentralized and digitalised container-based rearing systems developed in the framework of a research project in collaboration of Italian Cricket Farm Srl (Pinerolo, Italy) and OpacMare Spa (Torino, Italy). Larval production was carried out at the Experimental Zootechnical Centre of the University of Milan (Lodi, Italy).

**Figure 1:**
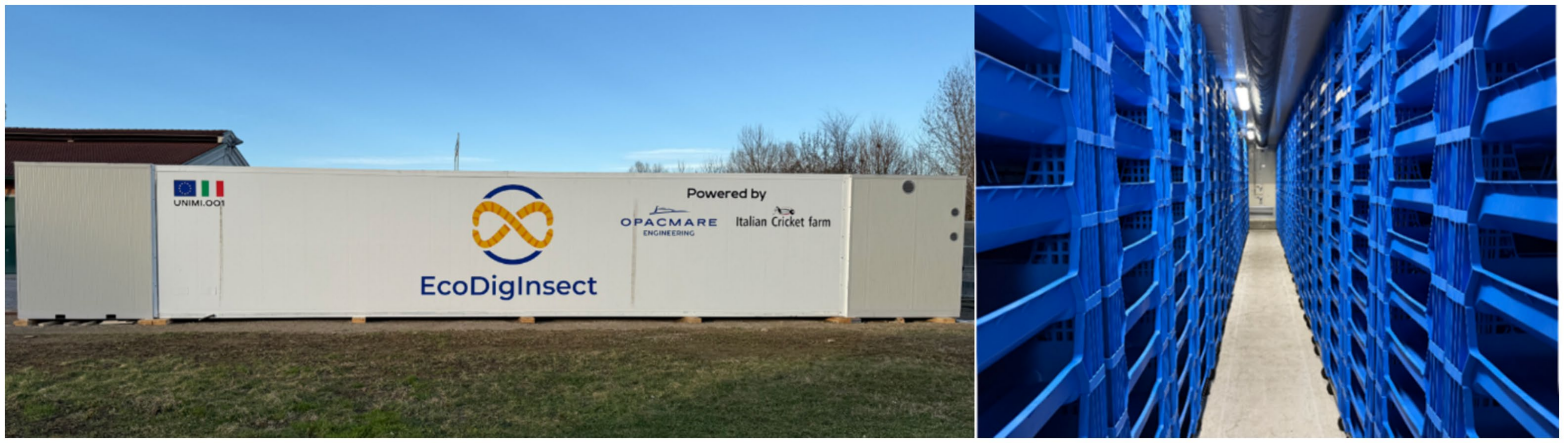
Experimental facility used for *T. molitor* larvae rearing – EcoDigInsect UNIMI001.

Particularly, larvae were reared in decentralized farming module specifically designed for insect production (EcoDigInsect – UNIMI001, Figure 1), equipped with 520 insect rearing trays, sensors for continuous monitoring and control of microclimatic parameters, including temperature, relative humidity, and gas concentrations (CO₂ and ammonia). Environmental conditions were maintained stable throughout the production cycle. In particular, temperature was set at 30 °C, while gas levels were constantly regulated to ensure optimal rearing conditions (CO_2_ < 1,500 ppm and NH4 < 30 ppm).

Larval feeding was based exclusively on wheat bran, which served as the rearing substrate for the entire production period. Hydration was provided following a progressive and time-controlled plan through an automated nebulisation system, with individual nebulisation units installed in each rearing tray, delivering multiple water administrations per day to ensure adequate hydration and environmental moisture (approximately 50–60% of relative humidity).

The production cycle lasted 9 weeks, from egg hatching to the larval–pre-pupal stage. All larvae used in the study originated from the same production batch. Once larvae reached the target developmental stage, they were harvested and stored in a refrigerated chamber at 10 °C for a limited period to reduce metabolic activity and temporarily halt further development. Before administration to piglets, larvae were gradually returned to room temperature for at least 4 h and supplied with wheat bran and hydration to allow acclimation, recovery of activity and improved motility.

Before administration to piglets, larvae were sieved to remove frass and residual substrate, then subdivided into plastic boxes and weighed to obtain the required amount for individual piglet supplementation.

### 2.2 Animal housing and experimental design

The trial was conducted at the Experimental Zootechnical Centre of the University of Milan (Lodi, Italy), following approval by the University’s Animal Welfare Organisation (OPBA; authorisation no. 41_2025). A total of 48 weaned piglets (Landrace × Large White; 28 ± 2 days of age), individually identified by ear tag, were allotted to four experimental groups using a stratified randomization procedure based on initial body weight and sex. This approach ensured balanced distribution of males and females (50% male, 50% female) and comparable initial body weight among groups (8.04 ± 0.88 kg). Each group consisted of 12 piglets housed in six pens (two piglets per pen) under standard environmental conditions (28 °C and 60–70% relative humidity), with temperature reduced by 1 °C per week starting 14 days after housing, for a total duration of 42 days.

Environmental enrichment was provided in each pen using jute ropes (two per pen), which were routinely replaced with new ones when worn or damaged and suspended inside the pen to provide manipulable materials, promote natural exploratory behaviours, and support animal welfare in accordance with Directive 2008/120/EC.

Two groups (CTRL15 and INS15) were fed a commercial 15% CP meal diet (DIET15), which contained no animal-derived meals protein sources. The same diet served as the base for DIET17: at the Experimental Zootechnical Centre of the University of Milan, 6.2 kg of soybean meal (44% CP) were added to 93.8 kg of DIET15 and homogenized for 20 min in a horizontal mixer to obtain a 17% CP formulation for groups CTRL17 and INS17. The ingredients and calculated chemical composition of both diets are presented in Table 1. All diets were offered *ad libitum*, were formulated to be isoenergetic, isonitrogenous and balanced for essential amino acids using Plurimix software and complied with NRC recommendations for post-weaning piglets (35). The commercial feed meal base diet was produced and supplied by Ferraroni S.p.A. (Bonemerse, Italy).

**Table 1:** Composition and main chemical characteristics of the experimental diets.

Ingredient, % (as-fed basis)	DIET15	DIET17
Barley meal	25.00	23.45
Wheat meal	16.45	15.42
Corn meal	10.00	9.38
Corn flakes meal	7.95	7.45
Dehulled soybean meal	5.50	5.16
Fermented soy protein concentrate	5.40	5.07
Biscuits meal	5.00	4.69
Wheat flakes meal	5.00	4.69
Dehulled barley flakes meal	4.00	3.75
Rapeseed meal, wheat meal, fermented beet molasses	3.50	3.29
Dried sugar beet pulp	1.50	1.41
Whey powder	1.50	1.41
Animal fat (lard)	1.50	1.41
Coconut oil	1.00	0.94
Fermented dairy products	1.00	0.94
Monocalcium phosphate	0.80	0.75
L-Lysine	0.70	0.66
Soybean oil	0.50	0.47
L-Threonine	0.26	0.24
DL-Methionine	0.20	0.19
Sodium chloride	0.20	0.19
L-Tryptophan	0.15	0.14
L-Valine	0.15	0.14
Vitamins and minerals premix	2.74	2.57
Soybean meal (44%CP)	-	6.2
Chemical composition % (as-fed basis)	DIET15	DIET17
Crude protein (%)	15.31	17
Ether extract (%)	5.42	5.17
Crude fibre (%)	3.49	3.47
Digestible energy (Mc/Kg)	3.42	3.43
K (%)	0.62	0.69
Cl (%)	0.39	0.37
Ca (%)	0.52	0.51
P (%)	0.46	0.47
Na (%)	0.15	0.14
S (%)	0.18	0.19
Mg (%)	0.11	0.12

Values are expressed as percentages on an as-fed basis.

Piglets in the INS15 and INS17 groups received a daily supplementation of 50 g per piglet (100 g per pen of two piglets) of live 9-week-old *T. molitor* larvae, reared on wheat bran and offered in a separate anti-spill feeder bowl. To provide an equivalent protein contribution, the CTRL15 and CTRL17 groups received 40 g per pen per day of soybean meal (44% CP). Water was available ad libitum through nipple drinkers (two per pen), and the health status of all piglets was monitored daily throughout the trial.

### 2.3 Chemical analysis and mineral determination of experimental diets

The proximate composition of the experimental diets, soybean meal and *T. molitor* larvae was determined according to AOAC methods (36), assessing dry matter (DM), ether extract (EE), crude protein (CP), and ash, while crude fibre (CF) was analysed only in the diets (37). Dry matter was measured by oven-drying samples at 65 °C for 24 h until a constant weight was achieved (AOAC 930.15). EE was obtained through petroleum ether extraction (AOAC 2003.05), while CP was analysed using the Kjeldahl procedure (AOAC 2001.11). Crude fibre (Weende) was determined following AOCS method Ba 6a-05, and ash content was quantified after incineration at 550 °C for 3 h (AOAC 942.05).

### 2.4 Zootechnical performance, faecal sample collection and health monitoring

Piglets were weighed individually on days 0, 7, 14, 21, 28, 35 and 42. Feed refuses were recorded weekly, and average daily gain (ADG), average daily feed intake (ADFI), and feed conversion ratio (FCR) were subsequently calculated.

**Figure 2:**
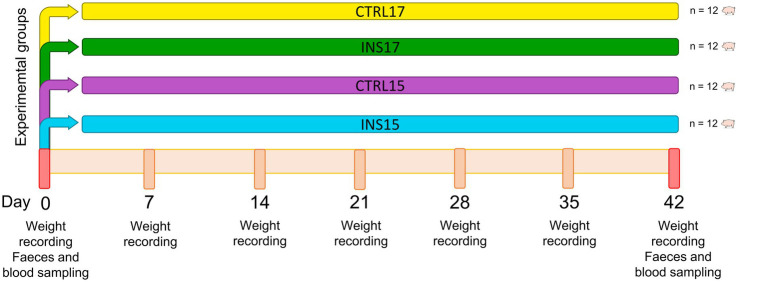
Schematic representation of the experimental design, showing the four experimental groups and the timeline of body weight measurements and sample collection.

Individual faecal samples were obtained on days 0 and 42 for diet digestibility evaluation and mineral content (Figure 2).

The health status of piglets was monitored daily using validated clinical scores according to Rossi et al. (38, 39). In particular, faecal consistency was scored based on consistency, using a four-point scale (0–3) considering scores ≤1 as normal, while values >1 were considered diarrhoeic. Faecal colour was also assessed with a three-level scale (0–2) with scores ≥2 were considered normal. Hair condition, respiratory and vitality scores also were assessed daily using a three-point scale (0–2) considering normal for a score = 0. For scoring purposes, each piglet was evaluated once per day. In addition, animals were visually inspected at least twice daily for more than one hour in total as part of routine health and welfare monitoring. However, repeated observations within the same day were not scored multiple times, in order to avoid artificial inflation of observations.

### 2.5 Live larvae administration and consumption time

The daily administration of 100 g of live *T. molitor* larvae per pen (50 g/pig/day) was carried out using an anti-spill feeder bowl. Live larvae were homogeneously distributed along the entire surface of the feeder to allow simultaneous access and consumption by both piglets housed in each pen, thereby limiting competition and dominance-related effects. In the CTRL groups, an equivalent protein contribution was provided simultaneously by offering 40 g of soybean meal (44% CP) per pen (20 g/pig/day). Consumption time was recorded at the pen level, as supplementation was provided per pen and individual intake could not be reliably quantified without altering housing conditions. For each administration, the time required for the piglets to completely consume the offered larvae or soybean meal was recorded per pen using a digital stopwatch. A maximum recording time of 10 min was applied. If the feed item was not fully consumed within this period, the 10-min cut-off was recorded as an indication of reduced interest.

To minimize bias and variability in timing, at least two trained operators performed supplementation and consumption-time measurements simultaneously, each monitoring multiple pens in parallel. This procedure ensured that supplementation was delivered to all pens within approximately 1 h, thereby standardizing the timing of administration across treatments and avoiding systematic differences related to order of feeding.

**Figure 3:**
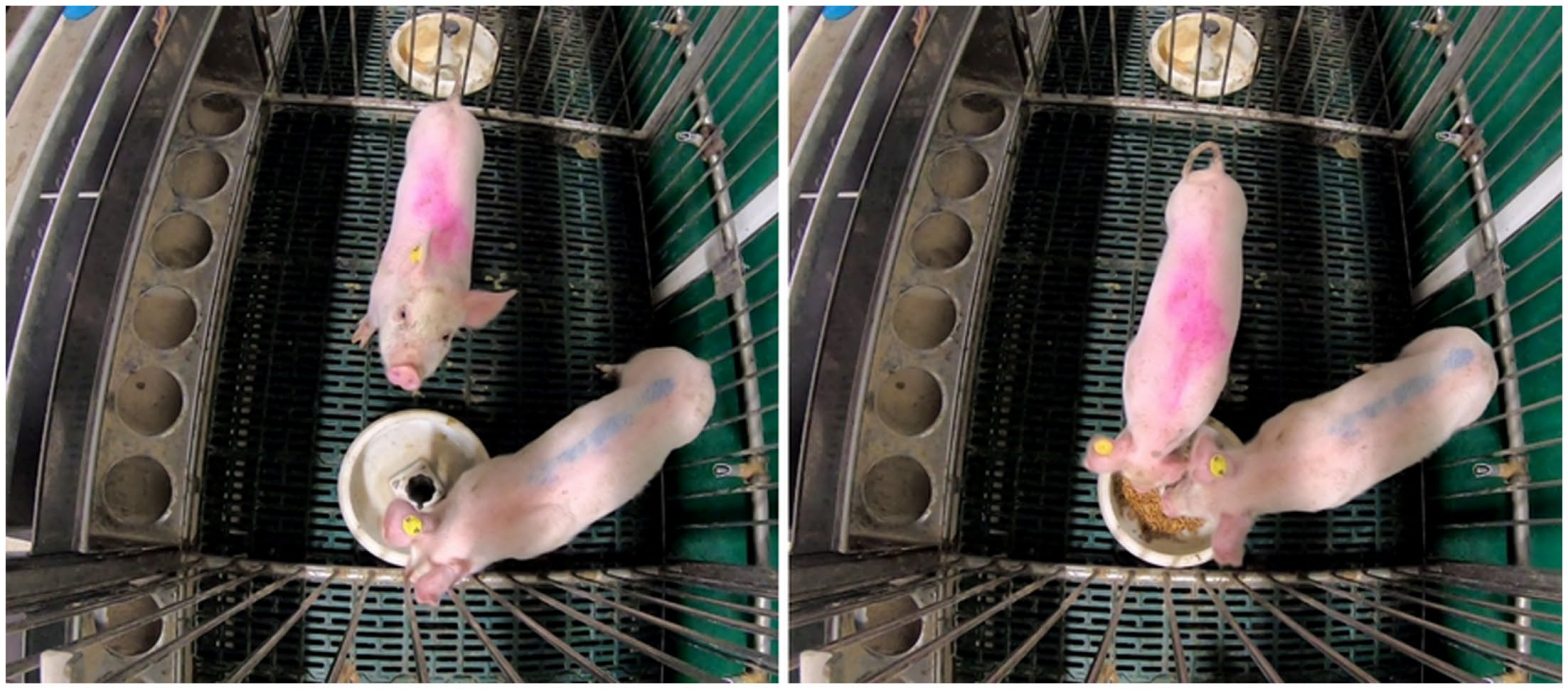
Feeder troughs used for live larvae or soybean administration in weaned piglets.

When residues of larvae or soybean meal were present after the recording period, they were removed and discarded, and a fresh portion of the respective supplement was offered at the subsequent administration. The feeder troughs used for live larvae and soybean supplementation (Figure 3) were maintained in the same position within each pen throughout the entire experimental period to avoid potential effects of feeder relocation on feeding behaviour and consumption time. The feeder troughs were cleaned each morning prior to the subsequent administration to avoid contamination or residue accumulation.

### 2.6 Nitrogen content and protein digestibility of faecal samples

Nitrogen content in faeces collected on days 0 and 42 was determined according to the Kjeldahl method (AOAC 2001.11). Apparent protein digestibility was calculated using the acid insoluble ash (AIA) marker technique applied to both feed and faecal samples (40). The apparent total tract digestibility (ATTD) of protein was then computed using the following equation:


$$\text{ATTD}\;(\%)=100\times [1-(\frac{\text{marker in feed}}{\text{marker in faeces}}\times \frac{\text{nutrient in faeces}}{\text{nutrient in feed}})]$$

### 2.7 Serum metabolites, oxidative status and mineral analyses

Individual blood samples were collected from jugular vein from each piglet on days 0 and 42 of the trial. Serum was obtained by centrifuging 10 mL vacuum tubes without anticoagulants at 3000 rpm for 15 min at 4 °C.

Clinical chemistry parameters were analysed only on samples collected at day 42 using a multiparametric autoanalyzer (ILab 650; Instrumentation Laboratory Company, Lexington, MA, United States). The following parameters were determined: calcium (mmol/L), alanine aminotransferase (ALT-GPT; IU/L), total protein (g/L), albumin (g/L), globulin (g/L), albumin-to-globulin ratio (A/G), urea (mmol/L), glucose (mmol/L), aspartate aminotransferase (IU/L), total bilirubin (μmol/L), total cholesterol (mmol/L), phosphorus (mmol/L), magnesium (mmol/L), and alkaline phosphatase (IU/L). Analyses were carried out at the Experimental Zooprophylactic Institute of Lombardy and Emilia Romagna (IZSLER, Brescia, Italy).

Serum oxidative status was assessed on samples collected at both day 0 and day 42 by measuring the antioxidant barrier capacity using the Oxy-Adsorbent test (Diacron Srl, Grosseto, Italy), according to the manufacturer’s instructions. Absorbance endpoints were read at 505 nm with a UV–Vis spectrophotometer (V630, Jasco GmbH, Pfungstadt, Germany), and results were expressed as μmol HClO/mL.

Mineral quantification in blood serum was carried out to compare the concentrations of trace elements including Na, Mg, Al, P, K, Ca, Cr, Mn, Fe, Co, Ni, Cu, Zn, As, Se, Mo, Cd, Tl, Pb. For sample preparation, 100 μL of serum were added to a 200 μL of 65% nitric acid (HNO_3_) and 100 μL of hydrogen peroxide in a 15 mL polypropylene tube. The mixture was vortexed and for dissolving, the tubes were set to 90 min at 60 °C in a heating system. After incubation the samples were cooling by adding 2,100 μL ultrapure water (RT) (41). Samples were then diluted 1:10 with a 0.3 M HNO_3_ solution in Milli-Q water. Concentration of elements was measured by ICP-MS (AGILENT 7850 ICP-MS). To check nebulization efficiency, a solution 200 μg L^−1^ of an internal standard solution (^45^Sc, ^72^Ge, ^89^Y, ^103^Rh, ^159^Tb, ^165^Ho) was introduced before the nebulizer in the sample flow. Analysis interferences were removed by a Collision Cell with a 5 mL min^−1^ He flow.

### 2.8 Statistical analysis

All data were analysed using GraphPad Prism (version 9.0) software. Prior to statistical testing, datasets were checked for normality through the Shapiro–Wilk test. Body weight, average daily gain, average daily feed intake, and feed conversion ratio were analysed using a linear mixed-effects model including treatment, time, and their interaction as fixed factors. The individual piglet was considered the experimental unit for BW and ADG, whereas the pen was considered the experimental unit for ADFI and FCR. When significant interactions were detected, pairwise comparisons were performed using Sidak’s post-hoc test.

The time required to consume the daily supplementation (live larvae or soybean meal), feed digestibility and antioxidant barrier status were analysed using a two-way repeated-measures ANOVA, considering treatment and time as fixed factors and repeated measurements taken on the same pens over the course of the trial.

For serum biochemical parameters and mineral concentrations measured at day 42, a one-way ANOVA was applied with treatment as the fixed factor. When significant effects were detected, Sidak’s multiple-comparison test was used to identify differences among groups.

For health-related daily data, the presence or absence of a given clinical condition was recorded once per day for each individual animal based on the daily clinical score (0 = absence; 1 = presence). Thus, for each parameter, each piglet generated a binary outcome for every day of observation.

Subsequently, daily records were summarised at the individual animal level across the entire experimental period. Each piglet was classified as positive for a given condition if a score of 1 was recorded on at least 1 day during the trial, or as negative if the condition was never observed.

The final dataset therefore consisted of the total number of animals per treatment group presenting or not presenting each condition, expressed as absolute counts and percentages. This approach ensured that each animal contributed a single independent outcome per health variable per day. The resulting dichotomous outcomes were then used to calculate absolute frequencies (number and percentage of affected animals) for each treatment group. Differences between observed and expected frequencies were assessed using the chi-squared test (*χ*^2^). When the overall chi-squared test indicated significance, post-hoc pairwise comparisons were performed using the Fisher’s exact test, applying Bonferroni *p*-value correction to limit the inflation of type I error. Results are presented as means ± standard error or standard deviation. Differences were considered statistically significant at *p* ≤ 0.05.

## 3 Results

### 3.1 Chemical analysis of diets and *T. molitor* larvae

Laboratory analyses confirmed the nutritional composition reported in Table 2, which highlights the difference in crude protein content between the 15 and 17% diets. Analysis of the diets showed protein levels of 15.87% for the 15% CP formulation and 17.83% for the 17% CP formulation. Nonetheless, the nutritional evaluation of the CTRL and INS diets confirmed that they were equivalent in terms of their main nutrient profiles.

**Table 2:** Chemical composition of experimental diets, *T. molitor* live larvae and soybean meal used in the experimental trial.

Analyte (%, as fed basis)	DIET15	DIET17	*T. molitor* larvae	Soybean meal (44% CP)
DM	93.34 ± 0.08	93.11 ± 0.08	35.60 ± 0.13	92.24 ± 0.05
CP	16.54 ± 0.76	17.83 ± 1.08	15.98 ± 0.16	42.95 ± 0.69
EE	5.15 ± 0.32	4.98 ± 0.04	6.95 ± 0.14	1.98 ± 0.10
CF	4.86 ± 0.28	4.81 ± 0.11	-	5.55 ± 0.54
Ash	5.58 ± 0.11	5.52 ± 0.05	1.46 ± 0.00	6.08 ± 0.00

DIET15, commercial diet formulated to contain a crude protein level of 15% as fed; DIET17, commercial diet supplemented with soybean meal to achieve a crude protein level of 17% as fed; DM, dry matter; CP, crude protein; EE, ether extract; CF, crude fibre. Results are expressed as mean ± standard deviation of three technical replicates for each parameter.

The compositional analysis of soybean meal and *T. molitor* (Figure 2) revealed clear nutritional differences between the two matrices. Soybean meal revealed 42.95% of CP, 1.98% lipids, 5.55% fibre, 7.76% moisture, and 6.08% ash. In contrast, *T. molitor* larvae showed a markedly higher moisture content (64.40%), 6.95% lipids, 1.46% ash, and 15.98% crude protein. Based on this protein level, each INS piglet received approximately 7.99 g of protein per head per day from the daily supplementation of live larvae.

### 3.2 Zootechnical performance

**Figure 4:**
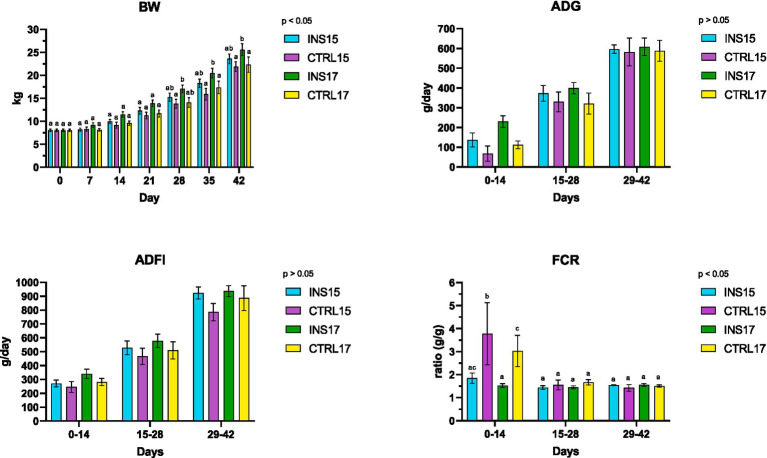
Growth performance of weaned piglets over the 42-day trial. Body weight (BW), average daily gain (ADG), average daily feed intake (ADFI), and feed conversion ratio (FCR) in piglets fed a 15% crude protein diet without larvae (CTRL15), a 15% crude protein diet with live *T. molitor* larvae supplementation (INS15), a 17% crude protein diet without larvae (CTRL17), or a 17% crude protein diet with larvae supplementation (INS17). Data are presented as means ± standard error. ^a,b,c^ Different lowercase letters indicate significant differences among groups within the considered period (*p* < 0.05).

Over the 42-day trial, body weight developed differently among dietary treatments (Figure 4). By day 28, the INS17 group showed a significantly higher body weight compared with CTRL15 (*p* < 0.05). At day 35, INS17 piglets were heavier than both CTRL15 and CTRL17 (*p* < 0.05). At the end of the experiment, piglets receiving live *T. molitor* larvae in the higher-protein diet (INS17) reached a significantly greater final body weight (25.58 ± 4.64 kg) than CTRL15 (21.90 ± 3.14 kg) and CTRL17 (22.33 ± 5.80 kg) (*p* < 0.05). At the same time point, INS15 piglets also displayed a significantly higher body weight compared with CTRL15 (*p* < 0.05). Interestingly, no significant differences were observed between INS15 and CTRL17, indicating that under a moderately reduced protein level (15% CP), the daily provision of live larvae supported growth to a level comparable with that of piglets receiving a higher-protein diet without larvae.

**Figure 5:**
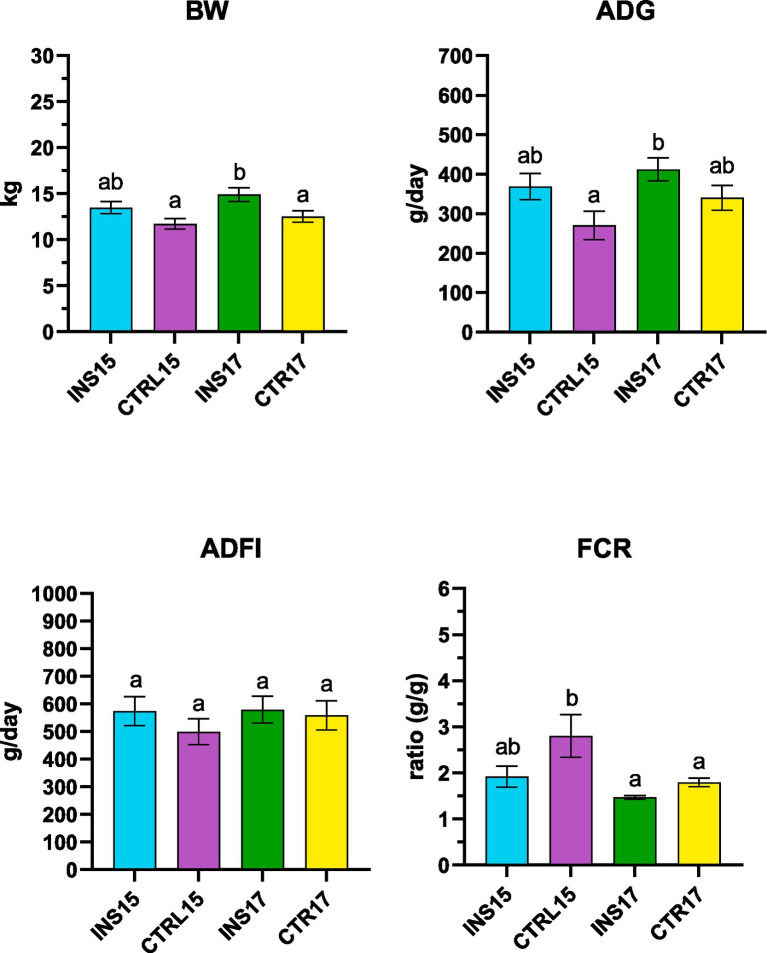
Growth performance of weaned piglets averaged for the 42-day trial. Body weight (BW), average daily gain (ADG), average daily feed intake (ADFI), and feed conversion ratio (FCR) in piglets fed a 15% crude protein diet without larvae (CTRL15), a 15% crude protein diet with live *T. molitor* larvae supplementation (INS15), a 17% crude protein diet without larvae (CTRL17), or a 17% crude protein diet with larvae supplementation (INS17). Data are presented as means ± standard error. ^a,b^ Different lowercase letters indicate significant differences among groups within the considered period (*p* < 0.05).

Patterns in ADG increased linearly for all groups throughout the 42-day trial (Figure 5). No significant differences were observed between treatments at individual timepoints. When considering the entire experimental period, the INS17 group displayed a significantly increased ADG (412.14 ± 33.56 g/day) compared with CTRL15 (270.26 ± 33.28 g/day) (*p* < 0.05).

Feed intake increased progressively with age and did not differ significantly among groups throughout the trial, with comparable values across treatments. FCR showed significant differences during the first 14 days: both INS17 and INS15 exhibited lower FCR values compared with CTRL17 and CTRL15 (*p* < 0.05), while no differences were observed among groups during the other periods of the trial. Moreover, considering the entire 42-day period, CTRL15 displayed a significantly higher FCR values compared to all other groups (*p* < 0.05).

### 3.3 Health status

**Figure 6:**
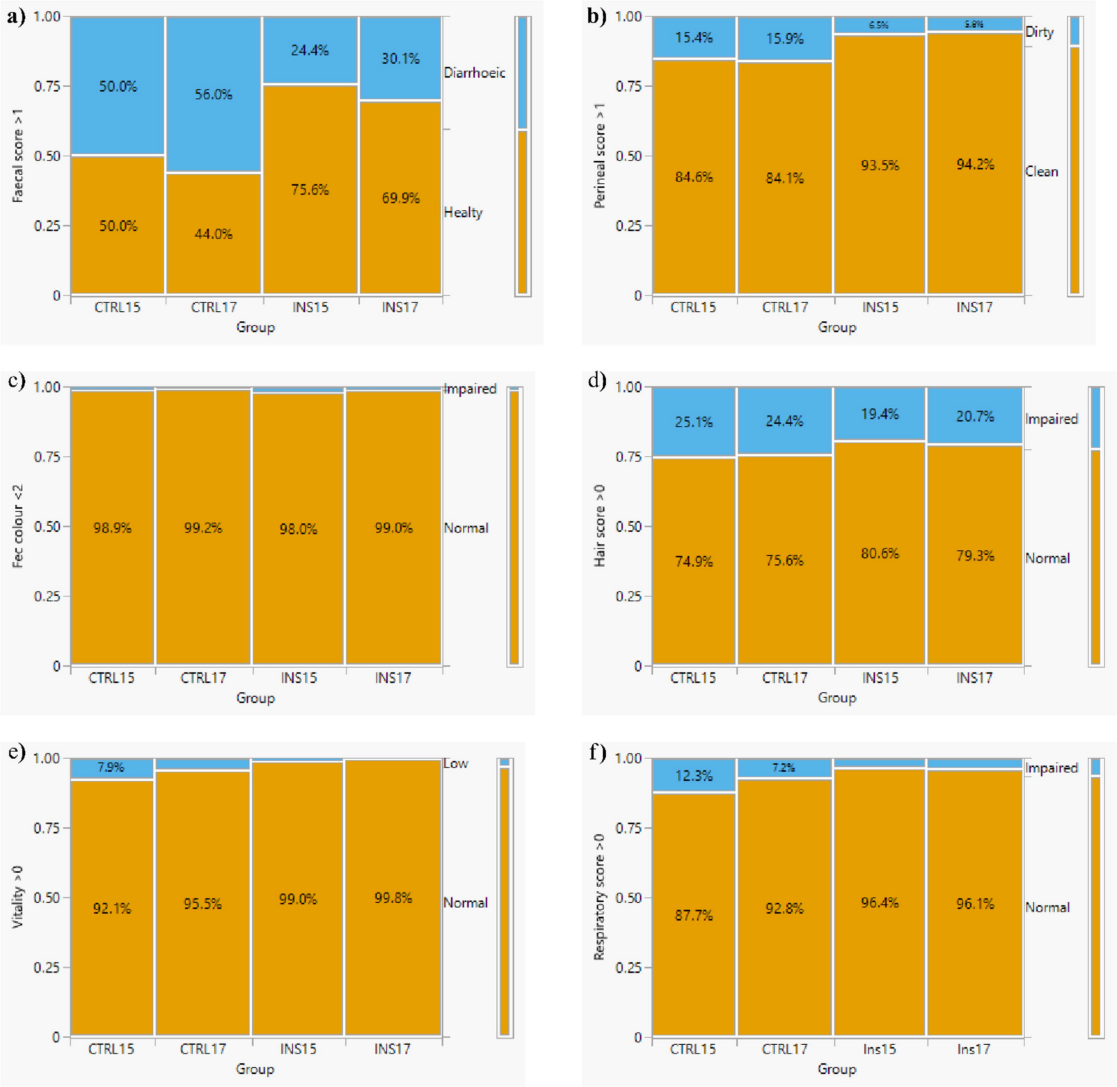
Health status evaluation through clinical scores for faecal score, viability, respiratory score, perineal score, hair score measured in weaned piglets receiving fed experimental diets with 15% or 17% crude protein levels, with or without live *T. molitor* larvae supplementation during the 42-days trial. **(a)** Faecal score, **(b)** perineal score, **(c)** hair coat colour, **(d)** hair score, **(e)** vitality score, **(f)** respiratory score. CTRL15, piglets fed the basal diet containing 15% crude protein without live larvae supplementation; CTRL17, piglets fed the basal diet containing 17% crude protein without live larvae supplementation; INS15, piglets fed the basal diet containing 15% crude protein supplemented daily with live *T. molitor* larvae; INS17, piglets fed the basal diet containing 17% crude protein supplemented with live *T. molitor* larvae.

The health status of the animals was monitored using four main parameters: incidence of diarrhoea, hair condition, vitality level, and the presence of respiratory disorders, all evaluated through validated scoring systems. Data analysis revealed statistically significant differences (*p* < 0.05) among groups (Figure 6; Table 3). Specifically, throughout the entire trial, the CTRL15 and CTRL17 groups exhibited a higher incidence of pathological scores compared with INS15 and INS17 groups. A particularly notable finding was the significant difference in the frequency of diarrhoea cases: 61 cases were recorded in the INS15 group and 65 cases in the INS17 group, compared with 124 cases in CTRL15 and 141 cases in CTRL17 (*p* < 0.01). Vitality scores were markedly higher in piglets receiving live *T. molitor* larvae, with both INS15 and INS17 showing significantly lower counts of low-vitality events compared with their respective CTRL groups (*p* < 0.05). Respiratory disorders followed a similar pattern. Both CTRL groups exhibited a substantially higher frequency of respiratory disturbances compared with INS groups, with CTRL17 showing the highest incidence (56 cases), followed by CTRL15 (37 cases). In contrast, INS15 and INS17 displayed markedly fewer respiratory issues (18 and 20 cases, respectively), with all comparisons reaching statistical significance (*p* < 0.05). Mortality and the need for therapeutic interventions showed a different situation. Two piglets from the CTRL15 group and one piglet from the INS15 group died due to respiratory disorders and further complications during the trial. Antibiotic treatments were required for three animals in CTRL15 and two animals in CTRL17, whereas only one piglet in INS15 required pharmacological treatment. Notably, no deaths and no antibiotic treatments were recorded in the INS17 group.

**Table 3:** Percentage of observations with altered clinical scores over the entire experimental period in piglets fed a 15% crude protein diet without larvae (CTRL15), a 17% crude protein diet without larvae (CTRL17), a 15% crude protein diet supplemented with live *T. molitor* larvae (INS15), or a 17% crude protein diet supplemented with live *T. molitor* larvae (INS17).

Score	Group				*p*-value
	CTRL15	CTRL17	INS15	INS17	
Faecal score (%)	12.84^a^	14.6^a^	6.31^b^	6.73^b^	<0.0001
Perineal (%)	3.53 ^a^	4.14 ^a^	1.61^b^	1.51^b^	<0.0001
Faecal colour (%)	0.25	0.20	0.50	0.25	0.3371
Hair (%)	5.75	6.35	4.84	5.40	0.0878
Vitality (%)	1.82^a^	1.16 ^a^	0.25 ^b^	0.05 ^b^	<0.0001
Respiratory (%)	2.82^a^	1.87^ab^	0.91^b^	1.01^b^	<0.0001

^a,b,c^ Different lowercase letters indicate significant differences among groups within the considered period (*p* < 0.001).

### 3.4 Consumption time of live larvae and soybean meal

**Figure 7:**
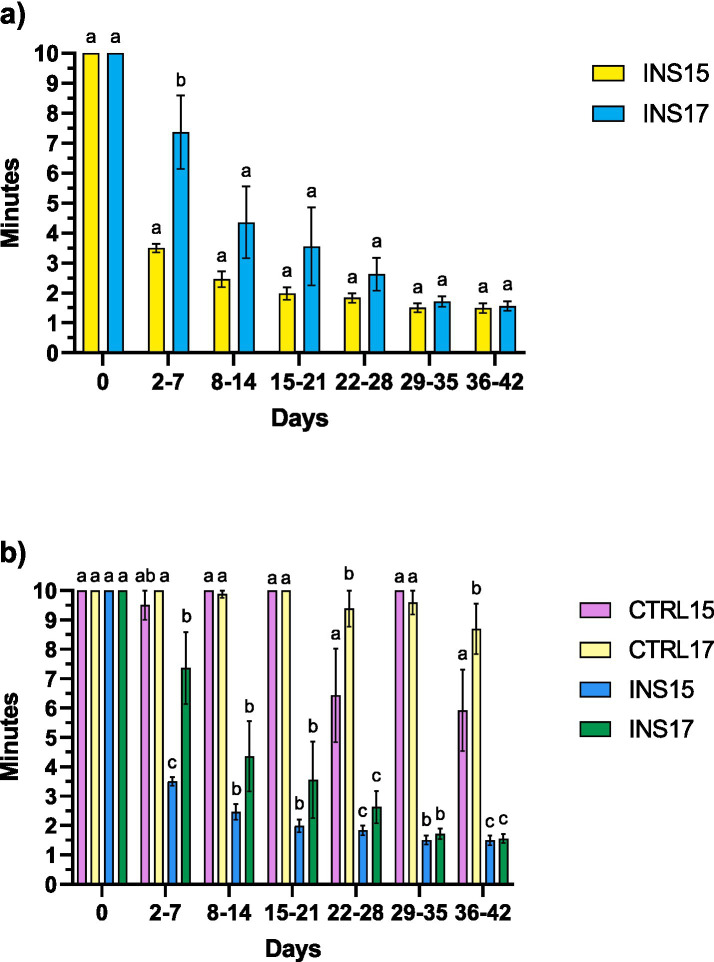
Consumption time of live *T. molitor* larvae or soybean meal in weaned piglets fed experimental diets with 15% or 17% crude protein levels, with or without live *T. molitor* larvae supplementation during the 42-day post-weaning period. **(a)** Time required for piglets receiving live larvae supplementation (INS15 and INS17) to consume the offered *T. molitor* larvae. **(b)** Time required for piglets fed soybean meal–based supplementation (CTRL15 and CTRL17) compared with larvae-supplemented groups (INS15 and INS17).

The average time required to consume the daily supplementation of live *T. molitor* larvae for INS groups and soybean meal for CTRL groups decreased progressively over the course of the trial (Figure 7). From the housing in the weaning facility, both INS15 and INS17 piglets started to consume the larvae. A significant difference emerged during days 2–7, when INS17 piglets required a longer time to complete consumption compared with INS15 (*p* < 0.001). From day 8, consumption time of larvae decreased steadily in both groups, with no further significant differences detected between INS15 and INS17. By the final weeks of the trial (days 29–42), both groups consistently consumed the supplementation within 2 min per pen, suggesting increasing familiarity and strong acceptance of the feed item over time. In contrast, soybean meal consumption in the CTRL groups showed a markedly different pattern. Across the entire trial, soybean meal frequently exceeded the 10-min cut-off, often remaining refused until the cleaning routine performed the following day. Although CTRL15 showed sporadic reductions in consumption time during the mid-trial period, values were highly variable among pens. Even in the final phase (days 36–42), the best average value recorded for CTRL15 remained reasonably high, at 5.92 min per pen, indicating lower interest and/or palatability compared to live larvae. The CTRL17 group exhibited an even lower level of acceptance: consumption time never dropped below 8.5 min per pen, consistently exceeding the threshold indicative of low palatability or interest.

Values represent the average time required per group to completely consume the daily supplementation. A maximum recording time of 10 min was applied; values reaching this cut-off indicate incomplete consumption within the observation period. Data are presented as mean ± standard error. Different lowercase letters indicate significant differences among groups within the considered period (*p* < 0.001).

### 3.5 Total and protein apparent digestibility of the experimental diets

Faecal dry matter content, used to calculate apparent total tract digestibility through AIA, was similar among groups at both the beginning and the end of the trial. At D0, faecal dry matter ranged from 23.94 ± 7.63% in CTRL17 to 27.33 ± 5.29% in CTRL15, with INS15 (26.48 ± 4.89%) and INS17 (26.14 ± 9.78%) showing comparable levels. A similar pattern was observed at D42, where faecal dry matter remained consistent across treatments (INS15: 27.13 ± 3.14%; CTRL15: 26.58 ± 3.83%; INS17: 25.16 ± 4.42%; CTRL17: 23.27 ± 5.19%).

Faecal nitrogen content also remained comparable among dietary treatments at both sampling times (0 and 42 days). At D0, %N on a dry matter basis showed similar values across groups (INS15: 1.00 ± 0.26%; CTRL15: 1.01 ± 0.19%; INS17: 0.85 ± 0.41%; CTRL17: 0.90 ± 0.46%), and the same trend was confirmed at D42, with no significant differences detected (INS15: 0.94 ± 0.11%; CTRL15: 0.92 ± 0.09%; INS17: 0.90 ± 0.16%; CTRL17: 0.86 ± 0.17%).

ATTD of DM, OM and CP evaluated at day 42 of the experimental trial showed comparable means for all considered treatments without highlighting significant differences (Table 4).

**Table 4:** Apparent total tract digestibility (ATTD) of experimental diets parameters measured at day 42 in weaned piglets receiving fed experimental diets with 15% or 17% crude protein levels, with or without live *T. molitor* larvae supplementation.

Parameter	Groups				*p*-value
	INS15	CTRL15	INS17	CTRL17	
DM (%)	81.29 ± 0.58	81.45 ± 0.68	81.44 ± 0.53	80.99 ± 0.50	0.9313
OM (%)	80.10 ± 0.61	80.27 ± 0.72	80.28 ± 0.56	79.79 ± 0.53	0.9321
CP (%)	76.16 ± 0.84	75.97 ± 1.77	76.73 ± 2.80	77.91 ± 0.47	0.8334

DM, dry matter; OM, organic matter; CP, crude protein; CTRL15, weaned piglets fed a 15% crude protein diet without larvae; CTRL15, piglets fed the basal diet containing 15% crude protein without live larvae supplementation; CTRL17, piglets fed the basal diet containing 17% crude protein without live larvae supplementation; INS15, piglets fed the basal diet containing 15% crude protein supplemented daily with live T. molitor larvae; INS17, piglets fed the basal diet containing 17% crude protein supplemented with live T. molitor larvae. Data are presented as means ± standard error. The number of experimental units was 12 animals per treatment for each parameter.

### 3.6 Serum metabolic profile, antioxidant barrier and mineral content

Analysis of serum biochemical parameters revealed no significant differences among groups, as shown in Table 5, which reports values at 42 days of trial. Serum antioxidant barrier did not differ significantly among treatments at either sampling time. At D0, mean Oxy Test values ranged from 277.77 ± 123.35 μmol HClO/mL in INS17 to 365.34 ± 118.06 μmol HClO/mL in CTRL17. Comparable values were recorded in INS15 (329.69 ± 134.21 μmol HClO/mL) and CTRL15 (312.80 ± 128.11 μmol HClO/mL). At the end of the trial, antioxidant capacity remained similar across groups. Values ranged from 308.75 ± 58.50 μmol HClO/mL in CTRL15 to 325.31 ± 33.27 μmol HClO/mL in INS17, with intermediate levels observed in INS15 (312.50 ± 42.43 μmol HClO/mL) and CTRL17 (318.04 ± 55.37 μmol HClO/mL).

**Table 5:** Serum biochemical parameters measured at day 42 in weaned piglets receiving fed experimental diets with 15% or 17% crude protein levels, with or without live *T. molitor* larvae supplementation.

Parameter	Groups				*p*-value
	CTRL15	CTRL17	INS15	INS17	
Total protein, g/L	57.5 ± 4.2	56.7 ± 5.0	57.9 ± 5.1	60.3 ± 4.6	0.3898
Albumin g/L	26.2 ± 3.8	27.0 ± 3.5	25.1 ± 3.1	26.6 ± 4.4	0.6669
Globulin, g/L	31.3 ± 5.3	29.7 ± 5.9	31.9 ± 5.0	33.7 ± 4.6	0.3996
A/G, g/L	0.9 ± 0.2	1.0 ± 0.3	0.8 ± 0.1	0.8 ± 0.2	0.3948
Urea, mmol/L	1.6 ± 0.5	2.0 ± 0.9	2.0 ± 0.7	2.4 ± 0.7	0.1498
ALT-GPT, IU/L	34.9 ± 6.0	35.1 ± 7.5	37.7 ± 7.2	37.9 ± 10.3	0.7411
AST-GOT, IU/L	34.1 ± 5.1	47.3 ± 12.6	35.8 ± 7.8	43.2 ± 26.5	0.2081
ALP, IU/L	213.3 ± 17.4	200.9 ± 41.6	177.5 ± 35.1	199.5 ± 60.5	0.2199
Total bilirubin, μmol/L	1.2 ± 0.7	0.6 ± 0.3	1.4 ± 0.6	1.3 ± 0.9	0.0931
Glucose, mmol/L	5.8 ± 0.6	5.9 ± 0.7	5.7 ± 0.8	5.3 ± 0.9	0.2948
Total cholesterol, mmol/L	2.5 ± 0.4	2.5 ± 0.2	2.4 ± 0.2	2.5 ± 0.4	0.6122
Calcium, mmol/L	3.4 ± 0.2	3.3 ± 0.1	3.3 ± 0.2	3.4 ± 0.3	0.2803
Phosphorous, mmol/L	2.9 ± 0.2	3.0 ± 0.3	3.0 ± 0.2	3.2 ± 0.3	0.1011
Magnesium, mmol/L	1.0 ± 0.1	1.0 ± 0.1	0.9 ± 0.1	0.9 ± 0.3	0.7634

CTRL15, piglets fed the basal diet containing 15% crude protein without live larvae supplementation; CTRL17, piglets fed the basal diet containing 17% crude protein without live larvae supplementation; INS15, piglets fed the basal diet containing 15% crude protein supplemented daily with live T. molitor larvae; INS17, piglets fed the basal diet containing 17% crude protein supplemented with live T. molitor larvae. ALT-GPT, alanine aminotransferase-glutamate pyruvate transaminase; AST-GOT, aspartate aminotransferase-glutamate oxaloacetate transaminase; ALP, alkaline phosphatase; A/G, albumin-to-globulin ratio. Data are presented as means ± standard error. The number of experimental units was 12 animals per treatment for each parameter.

Serum mineral concentrations measured at 42 days are reported in Table 6. No significant differences were detected among treatments for most of the analysed elements, including sodium, phosphorus, potassium, calcium, copper, zinc, arsenic, selenium, and molybdenum. Iron was the only mineral showing significant variation among treatments at day 42 of trial (*p* = 0.0235). Piglets in the INS17 group displayed the increased serum iron concentration (124.48 ± 39.70 μg/L) compared to INS15 (76.82 ± 37.25 μg/L).

**Table 6:** Serum mineral and trace element concentrations (μg/L) measured at day 42 in weaned piglets fed experimental diets with 15% or 17% crude protein levels, with or without live *T. molitor* larvae supplementation.

Minerals (μg/L)	Group				*p*-value
	INS15	CTRL15	INS17	CTRL17	
Na	133912.98 ± 15488.40	129297.58 ± 11588.33	128833.81 ± 18577.92	128746.49 ± 21004.10	0.8901
Mg	891.27 ± 127.44	842.54 ± 81.03	893.58 ± 160.10	930.97 ± 215.73	0.6681
Al	n.d.	n.d.	n.d.	n.d.	-
P	7896.90 ± 1022.88	7757.31 ± 710.09	7788.94 ± 1180.93	7598.16 ± 919.85	0.9576
K	8976.73 ± 1792.25	8367.72 ± 1037.40	8699.06 ± 1626.64	8799.94 ± 1573.90	0.8507
Ca	4772.02 ± 650.46	4690.25 ± 534.65	4549.70 ± 728.01	4551.68 ± 841.39	0.913
Cr	n.d.	n.d.	n.d.	n.d.	-
Mn	n.d.	n.d.	n.d.	n.d.	-
Fe	76.82 ± 37.25^a^	108.94 ± 32.99^ab^	124.48 ± 39.70^b^	105.75 ± 28.16^ab^	0.0235
Co	n.d.	n.d.	n.d.	n.d.	-
Ni	n.d.	n.d.	n.d.	n.d.	-
Cu	64.35 ± 10.06	61.17 ± 12.34	67.22 ± 15.72	63.86 ± 14.04	0.4336
Zn	19.34 ± 8.32	18.86 ± 9.87	15.77 ± 8.90	17.76 ± 12.01	0.8535
As	0.87 ± 0.15	0.87 ± 0.07	0.86 ± 0.10	0.83 ± 0.14	0.8435
Se	5.26 ± 0.92	4.73 ± 0.71^a^	4.92 ± 0.91	5.05 ± 1.10	0.6259
Mo	0.43 ± 0.15	0.52 ± 0.23	0.59 ± 0.42	0.59 ± 0.16	0.2793
Cd	n.d.	n.d.	n.d.	n.d.	-
Tl	n.d.	n.d.	n.d.	n.d.	-
Pb	n.d.	n.d.	n.d.	n.d.	-

Na, sodium; Mg, magnesium; Al, aluminium; P, phosphorus; K, potassium; Ca, calcium; Cr, chromium; Mn, manganese; Fe, iron; Co, cobalt; Ni, nickel; Cu, copper; Zn, zinc; As, arsenic; Se, selenium; Mo, molybdenum; Cd, cadmium; Tl, thallium; Pb, lead. CTRL 15%, piglets fed the basal diet containing 15% crude protein without live larvae supplementation; CTRL 17%, piglets fed the basal diet containing 17% crude protein without live larvae supplementation; INS 15%, piglets fed the basal diet containing 15% crude protein supplemented daily with live T. molitor larvae; INS 17%, piglets fed the basal diet containing 17% crude protein supplemented with live T. molitor larvae. n.d., not detectable. Data are presented as means ± standard error. The number of experimental units was 12 animals per treatment for each parameter.

## 4 Discussion

The post-weaning phase represents a major physiological challenge for piglets, as it is typically characterised by a marked reduction in feed intake that predisposes animals to impaired intestinal morphology, compromised barrier function and increased susceptibility to enteric disorders (42, 43). Strategies capable of stimulating early feeding behaviour are therefore essential to minimise post-weaning growth retardation and support adequate gut development. At the same time, precision-nutrition approaches are promoting lower crude protein diets to reduce nitrogen emissions and reliance on imported soybean meal, a feed ingredient increasingly associated with issues of availability, cost and sustainability in Europe (44). In this context, insects have emerged as promising alternative feed resources. Although several studies have investigated insect meals in pig nutrition (45), evidence on live larvae supplementation remains extremely scarce. The present study evaluated the effects of *T. molitor* larvae provided as a live supplement to piglets fed moderate or standard crude protein diets.

Larvae and soybean meal differed substantially in moisture and protein content. Nevertheless, the inclusion level of larvae resulted in a daily protein contribution comparable to soybean meal, ensuring a balanced availability of daily protein. Specifically, the estimated difference in crude protein intake between larvae- and soybean meal-supplemented piglets was −0.6 g per animal per day, indicating that the two supplementation strategies were functionally isonitrogenous under the conditions of the present study. In addition to protein, live larvae provided a higher lipid intake than soybean meal. However, the resulting difference in fat intake corresponded to an additional energy contribution of about 26 kcal per day (estimated using the modified Atwater coefficients) (46), which represents only a small fraction of the overall daily energy intake of weaned piglets. This minor difference is therefore unlikely to have substantially influenced growth performance on its own. Taken together, these considerations indicate that the observed effects of live larvae supplementation cannot be attributed solely to differences in protein or energy supply but rather reflect the combined contribution of adequate nutrient provision and other factors associated with live larvae supplementation, such as enhanced feed acceptance and behavioural stimulation.

Live larvae supplementation significantly influenced growth performance. Piglets in INS17 achieved higher body weights than both CTRL17 and CTRL15, suggesting a strong interaction between higher protein availability and larvae supplementation. Moreover, larvae-supplemented piglets exhibited improved feed efficiency, particularly during the early post-weaning period, as reflected by lower FCR values in the first weeks of the trial. Similar improvements in growth performance have been reported when insect-derived proteins were included in monogastric diets, particularly with *T. molitor* or *Hermetia illucens* meals, which enhanced feed efficiency and growth compared to conventional soybean meal-based diets (47, 48).

The mechanisms underlying this improvement are likely multifactorial. From a nutritional perspective, insects can provide essential amino acids with high biological value, potentially enhancing metabolic efficiency (49). The direct administration of live larvae, without thermal processing, may further increase amino acid bioavailability, as heat treatments are known to reduce lysine availability known as a limiting amino acid in pig nutrition, through Maillard reactions (50). In addition, the rapid and consistent consumption of larvae suggests enhanced palatability and reduced latency to the first meal post-weaning, which may attenuate the severity and duration of post-weaning anorexia. Earlier and more continuous feed intake is known to support intestinal development, preserve villus architecture and improve digestive capacity during the critical adaptation phase (51).

Remarkably, INS15 piglets, despite receiving lower dietary crude protein, achieved final weights comparable to CTRL17. This finding suggests that live larvae may partially compensate for lower protein intake not only through their nutrient contribution, but also by supporting gut functionality and overall health status, which may have contributed to and similar growth outcomes.

Finally, insects are known to contain antimicrobial peptides, which constitute part of their innate immune defence system (52). Although antimicrobial peptides and related bioactive compounds were not directly measured in the present study, their potential contribution to the improved health status and lower incidence of clinical disorders observed in larvae-supplemented piglets should not be excluded and will require further targeted investigation. Improved health conditions are closely linked to better feed efficiency, as inflammatory and disease challenges are known to impair nutrient utilisation and growth (34). Taken together, these factors provide a plausible explanation for the enhanced growth performance and feed efficiency observed in piglets receiving live larvae supplementation.

Improved FCR during the initial 2 weeks in INS groups therefore suggests faster stabilisation of digestive processes and better nutrient utilisation in animals exhibiting stronger early feeding behaviour. This improvement should not be interpreted as the result of a single mechanism, but rather as the outcome of the interaction among several complementary factors. Continuity of feed intake appears to play a central role, as piglets that initiate feeding earlier and maintain more consistent ingestion patterns (“early eaters”) are known to display better health status and growth efficiency during the post-weaning period (4, 53). The supplementation of live larvae likely enhanced feed acceptance during a period characterized by post-weaning anorexia, thereby supporting intestinal integrity and reducing the metabolic costs associated with gut adaptation. In addition, live insect supplementation may have acted as a form of feeding enrichment, potentially attenuating stress-related energy expenditure, although this aspect warrants further investigation.

Live larvae likely acted as a multimodal sensory stimulus, providing visual, olfactory and tactile cues that enhanced exploratory behaviour and feeding motivation. From a behavioural standpoint, larvae movement and palatability likely enhanced feeding motivation, resulting in earlier and more consistent feed consumption. This behavioural stimulation has been observed in other species offered live insects, such as poultry, where increased exploratory behaviour translated into improved health and welfare status (54–56). Such stimulation may have increased the frequency of feeder visits and promoted earlier and more frequent meals, particularly in the immediate post-weaning phase. In pigs, early and frequent feeding immediately after weaning is critical to prevent anorexia, villus atrophy, and intestinal inflammation (13).

Piglets receiving live larvae may have promoted an early feed engagement, which likely supported mucosal development and improved nutrient utilization efficiency. These findings align with existing evidence that early and frequent feed consumption alleviates villous atrophy and promotes intestinal integrity during weaning (57–59). Furthermore, studies with insect-derived chitin and antimicrobial peptides have reported modulation of gut microbiota and barrier function, potentially contributing to improved digestive stability (19, 60). However, these mechanistic pathways were not directly assessed in the present study and should therefore be interpreted as hypotheses to be tested in future research.

Consumption time was markedly different between treatments. Live larvae were consumed rapidly from the first days, with INS groups displaying a consistent decrease in consumption time until stabilising at 1–2 min during the last 2 weeks, indicating strong palatability, and rapid adaptation to this novel feeding strategy. This observation supports the idea that live larvae are perceived as a highly stimulating feed source, combining both sensory attractiveness and nutritional reward. Larvae’s natural movement, texture, and odour likely triggered innate exploratory and feeding behaviours in piglets, which are particularly active during the post-weaning adaptation phase (61, 62).

In contrast, soybean meal supplementation in CTRL groups was weakly accepted: consumption frequently exceeded the 10-min cut-off and was often refused until the next day. The relatively low palatability may be related to the minimal sensory complexity of a dried meal and the absence of visual or tactile stimuli capable of eliciting investigative feeding behaviour. It should also be acknowledged that soybean meal represents a nutritional control rather than a behavioural analogue of live larvae. Therefore, differences in consumption time between treatments should not be interpreted as a direct comparison of enrichment value. In the present study, consumption time is primarily intended as an indicator of relative palatability and feeding motivation. The potential enrichment effect of live larvae, linked to their sensory and dynamic characteristics will require future dedicated behavioural and welfare assessments. The higher acceptance of larvae therefore likely contributed directly to the improved performance observed in INS groups, by promoting more consistent and earlier feed intake.

Beyond their biological relevance, these results have important practical implications. High palatability, as objectively confirmed by consumption time, is a key requirement for successful on-farm application of insect-based feeding strategies. The use of live larvae allows the elimination of processing steps such as drying and milling, which substantially increase production costs due to high energy requirements (63). In this regard, previous studies on insect meals production have shown that drying represents one of the most energy-demanding and cost-relevant steps in insect meal production. In this regard, a study by Kröncke et al. (64) reported that the cost of oven drying insects is approximately 3.24 €/kg, followed by freeze-drying at 2.88 €/kg and rack-oven drying at 0.67 €/kg. Although these values vary with time, scale, experimental conditions, and energy costs, they offer an order-of-magnitude of the impact of thermal processing on the overall production cost of insect meals. This aspect is particularly relevant, as economic constraints currently represent one of the main limitations to the adoption of insects in swine and livestock nutrition.

Moreover, the possibility of producing larvae in decentralised, modular rearing systems could further reduce costs associated with transport and logistics, especially when integrated into local circular-economy models. Although a precise economic evaluation was beyond the scope of the present study, the strong acceptance observed suggests that live larvae may represent a cost-efficient and practically viable alternative to processed insect meals, warranting further investigation into their economic sustainability at farm level.

Comparable findings have been reported in other species, where live insect supplementation enhanced feeding motivation and welfare. In poultry, for example, the inclusion of live *Hermetia illucens* or *T. molitor* larvae increased pecking activity and reduced stress-related behaviours (55, 65). These data suggest that the environmental enrichment effect of live larvae feeding may extend beyond simple palatability, providing cognitive and emotional stimulation that supports feed intake, health and welfare (55). Taken together, these results indicate that the introduction of live larvae during the weaning transition not only enhances feed acceptance but also aligns with the animal’s natural exploratory behaviour, leading to improved intake dynamics and, ultimately, better growth performance.

Health scores were consistently improved in larvae-supplemented piglets. INS groups showed lower diarrhoea episodes, fewer respiratory disorders, and better vitality scores compared with CTRL animals. The difference in diarrhoea frequency was particularly pronounced and is consistent with improved intestinal stability and early feed intake, which reduce the risk of post-weaning dysbiosis (66). Similar reductions in diarrhoea incidence have been reported in piglets receiving insect-derived meals, suggesting that insect components such as chitin and antimicrobial peptides may exert protective effects on gut health (67, 68). Chitin and its derivative chitosan are known to modulate the gut microbiota by promoting beneficial bacterial populations (e.g., lactic acid bacteria) and inhibiting opportunistic pathogens such as *E. coli* and *Clostridium* spp. (69–73). While these properties have been described in previous studies, their specific contribution under the conditions of the present experiment cannot be confirmed, as no microbiome or immune-related analyses were performed. These bioactive components, together with early feed intake stimulation, may plausibly contribute to the enhanced gut stability observed in INS groups.

INS17 showed neither mortality nor antibiotic treatments, whereas CTRL groups exhibited a higher incidence of disease and required more therapeutic interventions. Comparable improvements in health status and reduced need for treatments have been observed in weaned pigs fed insect meals, particularly *Hermetia illucens*, which enhanced mucosal immunity and reduced inflammatory markers (74). These findings strengthen the role of early feeding behaviour and sensory enrichment in promoting resilience during post-weaning stress.

It should be acknowledged that the present study was not specifically designed to investigate the mechanistic pathways underlying the observed improvements in performance and health. Future studies should specifically address these aspects through targeted approach to elucidate the biological mechanisms potentially involved in the responses observed following live larvae supplementation.

From a broader perspective, the combination of nutritional and environmental enrichment offered by live larvae may create a synergistic effect, stimulating feed intake while supporting intestinal and immune homeostasis. Such dual benefits are highly relevant for antibiotic reduction in pig production systems, where the capacity to maintain gut health through functional nutritional strategies is increasingly critical (21, 73, 75, 76).

Neither crude protein level nor larvae supplementation influenced apparent total tract digestibility or faecal nitrogen excretion. Digestibility remained stable across groups at day 42, indicating that a moderate reduction in dietary crude protein does not impair digestive efficiency when the amino acid profile is properly balanced. This observation aligns with recent evidence showing that lowering crude protein in piglet diets, when compensated with supplemented amino acids, maintains nutrient digestibility and growth performance while reducing nitrogen emissions (77).

It should be noted, however, that apparent digestibility was assessed only at day 42, when piglets probably had largely completed their physiological and microbial adaptation to solid feed. Therefore, the absence of differences at this time point does not exclude the possibility that transient effects on digestive efficiency or nutrient absorption may have occurred during the early post-weaning phase, which was not directly evaluated in the present study.

In recent years, increasing attention has been given to the role of dietary chitin from insects. Chitin has been reported to slightly reduce protein and fat digestibility only at higher inclusion levels or when poorly deacetylated (78). At low concentrations (<1%), however, chitin may even exert prebiotic and immunomodulatory functions without compromising feed efficiency (79). Therefore, the improvements in growth and health observed in INS groups could be partially attributable to functional effects of larvae supplementation, such as enhanced gut integrity, microbial modulation, and immune stimulation, rather than changes in digestive capacity. Moreover, the absence of negative effects on apparent digestibility following larvae supplementation suggests that the chitin content of *T. molitor* larvae was below the threshold (1–1.5% of the diet) at which interference with nutrient utilization becomes significant (79).

Overall, these findings support the concept that the benefits of live larvae supplementation may derive from a combination of behavioural stimulation and functional properties of the larvae, including earlier feed engagement and improved intake continuity during the critical post-weaning phase. In addition, we hypothesize that a potential prebiotic role of chitin may have contributed to the observed effects.

Serum biochemical parameters remained within physiological ranges and did not differ among treatments, indicating that both experimental diets and larvae supplementation supported normal metabolic function.

Serum antioxidant capacity did not differ among groups. This stability aligns with the physiological tendency to maintain redox homeostasis in the absence of strong oxidative stimuli. As noted, serum markers may not fully reflect local intestinal redox adaptations. However, the absence of systemic oxidative impairment confirms that live larvae supplementation was safe and did not induce metabolic stress.

Most minerals did not differ among treatments, confirming overall mineral homeostasis. The moderate increase in serum iron in INS17 may be linked to higher feed intake or improved absorptive efficiency, though values remained within physiological ranges. The current findings showing a moderate, non-pathological increase in serum iron in the INS17 group can be interpreted within the broader context of the known interactions between chitin and iron absorption. Chitin, a structural polysaccharide abundant in insect exoskeletons, is recognized for its ability to bind transition metals, including iron (80). This chelating capacity has been confirmed in both environmental and structural studies, where chitin and chitosan efficiently sequester ferric ions or form stable complexes with iron (81, 82). Such properties have led some authors to hypothesize that chitin may impede iron bioavailability when consumed in diets rich in insect biomass. Iron absorption from *T. molitor* larvae, either with native or reduced chitin content, was found to be comparable to that from maize porridge, suggesting that dechitinization does not significantly alter fractional iron absorption (80). Similarly, in studies using *Acheta domesticus* (house crickets), iron absorption was relatively low but consistent with non-heme sources, with *in vitro* tests confirming that chitin and chitosan reduced iron bioaccessibility mainly through binding rather than intestinal inhibition (83). These findings suggest that while chitin has a measurable affinity for iron, its biological effect is modest under typical dietary conditions. Moderate chitin inclusion may even have indirectly positive effects on nutrient utilization via enhanced gut health, increased villi length, and microbiota modulation (79).

Taken together, the results demonstrate that daily supplementation with live *T. molitor* larvae could promote early feed intake, improve performance, and support health without compromising digestibility or metabolic status. Notably, larvae allowed piglets fed a 15% crude protein diet to reach performance comparable to those fed 17%, suggesting that stimulation provided by live insects may facilitate more sustainable dietary crude protein reduction strategies.

These findings align with the growing interest in insects as functional feed ingredients and highlight the potential of live larvae to act not only as nutrient sources but also as a form of environmental enrichment that enhances piglet resilience during weaning. By supporting early feed intake and overall health, this strategy may also contribute to reducing the need for antibiotic treatments, in line with current efforts to mitigate antimicrobial resistance.

## 5 Conclusion

In conclusion, supplementation with live *T. molitor* larvae showed to be an effective strategy to support piglet adaptation during the post-weaning phase. Live larvae markedly enhanced feed motivation and early intake, resulting in improved growth performance and feed efficiency, particularly during the initial weeks after weaning. Notably, piglets receiving larvae supplementation under a 15% crude protein diet achieved growth performance comparable to those fed 17% crude protein diets. Live larvae supplementation was also associated with improved health status, including reduced diarrhoea incidence and lower antibiotic treatments, without adversely affecting nutrient digestibility, serum metabolic profile, antioxidant capacity or mineral homeostasis. Overall, these findings suggest that live insects may serve not only as innovative nutrient sources but also as functional feed capable of improving piglets’ resilience during weaning. This approach may support precision feeding strategies by lowering the total dietary protein while supporting animal performance and health.

## Data Availability

The original contributions presented in the study are included in the article/supplementary material, further inquiries can be directed to the corresponding author.
